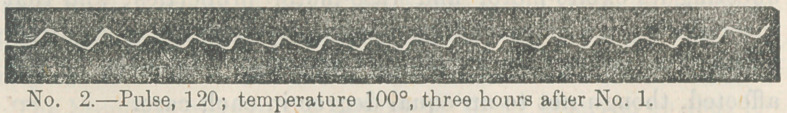# The Bromides

**Published:** 1869-05

**Authors:** Thomas G. Williams


ARTICLE XVIII.

### THE BROMIDES.

AN INAUGRUAL THESIS, PRESENTED TO FACULTY OF CHCAGO MEDICAL COLLEGE, 1869.

BY THOMAS G. WILLIAMS.

The bromides constitute a numerous and an important class of compounds. As medicinal agents they have of late attracted an unusual degree of attention. Although many of them are of great value, but a few have as yet been sufficiently investigated to justify us in feeling that we know much with regard to their medicinal efficacy. Those which have most attracted and are now attracting the attention of the profession are the bromides of sodium, potassium, ammonium, and to a certain extent quinia. With regard to the first three mentioned, the bromide of sodium is generally considered by all observers as of much less efficacy than the other two; and for this reason we will give it but little attention. The bromide of quinia has been tried in a few instances with apparent success; but as yet only a few observations have been made, and all that can be now said about it will be mere conjecture. As to the bromides of potassium and ammonium this is not true, for they have been used extensively, and many careful and comprehensive experiments have been made on them.

The bromide of potassium seems to be the favorite article in all the recorded observations, but for what reason I cannot tell. I am satisfied that the bromide of ammonium is the more useful as well as the more pleasant of the two, and deserves greater attention than is usually given to it.

After careful trial of the two, in many different circumstances, I do not hesitate to say that as a remedial agent it as far surpasses the bromide of potassium as the latter does the bromide of sodium. Why this is so I cannot explain, except in the same manner as some attempt to explain why the bromide of potassium is more efficient than the bromide of sodium. They say that the salts of potassium are all more efficient than the corresponding ones of sodium, therefore the bromide of potassium is the more powerful. For a similar reason the bromide of ammonium is more powerful than the bromide of potassium. We know from experience that most of the salts of ammonium are more decided in their effects than the corresponding ones of potassium. A good understanding of either of them, however, will suffice as a standard with which to judge the others; for they are similar in action, differing only in degree.

It has been a matter of considerable discussion whether the action is peculiar to the combination or to one of the elements. Some think that the base is the active ingredient, others that bromine is, while the majority of writers conclude that the salt as such, is the agent which produces the peculiar effect. Who ever saw any of the other salts of potassium or ammonium produce the effects derived from these under consideration; or who ever thought bromine produced such effect? It seems to me to be impossible to account for the phenomena produced when these salts are given, except to ascribe to them, as such, the peculiar virtue. Dr. Bill claims that double decomposition takes place with chloride of sodium as soon as it enters the blood, and that we virtually give bromide of sodium, which he regards as a substitute for chloride of sodium, with a chloride of the base given. This statement no one can hardly believe, after he has fairly studied the matter. We shall be better prepared to see the error of this statement as we progress.

We may, I think, without doubt regard the salt itself as the active agent, and that it continues as the salt given until its elimination from the body.

The thing to be done, now that these points are settled, is to find out how they act and the manifestations of their action. How do medicines act? This is a question yet unanswered; but the manifestations resulting from their actions are capable of being studied and described. It is not an easy thing to observe and to describe rightly these manifestations, as we may readily infer from the variety of opinions held concerning the different articles of the *materia medica.* This we shall see to be specially true with regard to the subject under consideration, before we have finished.

Most views entertained concerning the manner in which substances act when given as medicines, or taken in poisonous doses, have been extremely vague and unsatisfactory. They are often regarded as warriors, intelligent and ready to battle with and drive out of the system whatever does not belong there. Our text-books are so written that a student, in studying them for the first time, unavoidably comes to entertain these absurd views. It seems as though men dare not investigate this subject, but feel satisfied in believing that it is beyond the human power to explain. But this feeling is fast wearing away as organic chemistry unfolds to our observation things before inexplicable. Great minds are becoming unsatisfied with the old theories, and are looking about them for explanations in accordance with fixed and known physical laws.

Some writers have hinted that the bromides are efficacious, from their property of diminishing metamorphosis of tissue; a few claiming that it exercises no elective action, while others claim that it does, acting only on nerve tissue. McElroy has written a very ingenious article on this subject. It deserves a careful consideration at least, for I think it opens up a new course of investigations, even if the views therein contained are not true. He recognizes two grand processes as going on in the system, and terms them nutrition or constructive metamorphosis and oxidation or destructive metamorphosis. Medicines, he says, may promote or retard either of these processes, thus giving us four classes of action to study. To illustrate, he says that chloroform arrests oxidation; strychina promotes it; calomel retards nutrition, and promotes oxidation. The bromides, he further says, owe their effects to the property of promoting the oxidation and consequent elimination of effete material, or that which is below the natural standard of development. He claims that these views are true beyond a doubt. How true they may be I am not prepared to say; but this I do know, that he has done much towards explaining how medicines should be studied. It will be impossible to say at present how the bromides do act; whether it is, as the ancients believed and indeed some of the modern observers as well, that they act as an intelligent creature, driving out the disease, or as a catalytic agent, acting by its mere presence; whether it diminishes metamorphosis of tissue, or whether it is in the manner explained by McElroy, which we have just noticed. But this we do know, that they act somehow, and give rise to peculiar manifestations, and are often of inestimable value to the physician in the cure of many diseases.

We will devote the remainder of our attention to the study of these effects. It might appear at first thought that this is easy. But we shall find, as we progress, that it is not so simple after all. There are many things, in the way of making observations, which will lead to the same conclusion under different circumstances. Our physiologists are so various in their views of life action that no real standard can be chosen from which to judge. The terms used by different observers are not understood alike by each. The circumstances under which experiments are conducted vary the results to a great extent. Indeed, it is not to be wondered at that men differ greatly, when we take everything into consideration.

Having derived splendid results from the use of the bromides, I was led to investigate the views entertained by different ones in regard to them. Though recently brought into notice, they have attracted very great attention. I was somewhat astonished at the variety of opinions adduced by different investigators. In order that we may the better reach the right conclusion, which we must do or our prescribing will be but mere empiricism, let us examine the conclusions reached by some of the best observers.

Eulenburg and Guttman, German observers, after elaborate experiments on frogs and some of the other lower animals, conclude that it (the bromide of potassium) is rapidly absorbed into the blood, that it is an intense cardiac poison, and that it paralyzes muscular and nerve action. Nervous and muscular irritability are both destroyed by its prolonged use. Its therapeutic action is anti-spasmodic, anti-convulsive, and anæsthetic. It has no direct hypnotic or narcotic action.

Ladorbe, a Frenchman, thinks that it acts only on the spinal cord, diminishing especially reflex action; that it has no effect on the circulation, mental action or sensation. Voluntary motion remains unaffected.

Damourette and Pelvet believe, as those above, that it acts through the circulation. They claim that it has no elective action, but that it acts on all tissues alike. The action is sedative, and is produced by direct contact. Nerve force yields most readily and heart action last. Therapeutic action is anæsthetic and a general sedative, acting specially on no particular function, but on all alike, and that by actual contact. Brown-Séquard terms it a nerve sedative, having no special influence otherwise. He says that it is indicated only where a sedative effect on the nervous system is required.

Bartholow, of Cincinnati, regards it as a powerful irritant to mucous membrane of the stomach and a vascular and nerve sedative. Therapeutically it is a disinfectant, escharotic, hypnotic, and sedative; but not indicated in cerebral congestion. Dr. Begbie, of Georgia, thinks it is a nerve sedative and a hypnotic, useful only in diseases of nervous origin. Dr. Bill, U. S. A., has made several series of investigations to decide on its physiological effects, and some of his conclusions are interesting. As before noticed, he says that we virtually give the bromide of sodium, which is powerless as a remedial agent. His conclusions are, that only the peripheral nerves of mucous membranes are affected in the least, for as soon as it enters the blood it is changed into two powerless salts by double decomposition. If we gave sufficient to neutralize the chloride of sodium, we might get the peculiar effect of the bromide elsewhere from its local action. We get its peculiar action on the digestive tract and also on the urinary apparatus. It does not appear to me how this is possible, from his description, but it does seem that he has complicated things considerably. His article is long, but contains many peculiar inconsistencies. For instance, he speaks of its diminishing the elimination of carbonic acid gas, but as not affecting its formation in the system. It therefore acts as a soporific, by virtue of the retained carbonic acid gas. He also speaks of its peculiar action on the urinary organs, as well as many other things, which compel us to ask how this is possible, since none enters the circulation. It cannot be the effect of the bromide of sodium, for he tells us that this salt is like the chloride of sodium in its effects. As for the chloride of potassium, we know that it has no such effect. Thus we are almost compelled to doubt the truthfulness of his deductions, from the incongruities he has produced. However, in view of all this there is much practical information to be derived from the study of his article. The views of many others might be produced, if it were necessary. However, I think that I have noticed a sufficient number to enable us to form an opinion of the obscurity in which we are left when we seek an explanation of the "*modus operandi*" of the bromide of potassium, from the literature of the present day. We have seen that much difference of opinion exists. Some will say unhesitatingly that we get only a local action on the peripheral nerves of digestive and urinary tracts; others that it has a special action on nerve centres, or that the spinal cord is affected, or the nerves of sensation, excito-motor or vaso-motor nerves. Some say that it makes no distinction with regard to the tissues it selects; and in fact we may say that all manner of opinions are extant, even to saying that it has no effect at all. I could not be satisfied from what I could find by reading, so I determined to make observations for myself.

In order to make no mistakes and to avoid some of the sources of error, I made several experiments both upon myself and upon others. The results were so similar that I do not think it best to enter into a detailed account of each, but to produce what will be of use to us in this investigation. My first object was to find the effect of its prolonged use on the secretions, nutrition, and nervous system. From my reading I was led to think that circulation was but little, if at all, affected. I therefore had made no preparation for observing the effect on circulation and temperature. Some claim, also, that the effect is rendered peculiar, by the amount given. Not believing the notion that some advocate, of the different results derived from medicines when given in large or small doses, I made observations in order to satisfy myself on the action of the bromides in this respect. I found only a difference in degree of action between large and small doses. I do not understand why such a notion has become so common with our profession. We have various articles, for instance, which in small doses are called stimulants, but in larger doses they are called sedatives. I think it must be that we do not know what constitutes stimulation and what sedation. However that may be, we get a similar effect from the article under consideration under all circumstances, differing only in degree. The article which I used was manufactured by Powers and Weightman, Philadelphia. I took 10 and 15 grains three times a day for a considerable time. The results were so much like the following that I will not notice them here. When I had fully recovered my normal condition, I commenced taking ℈ij. 4 times a day and carefully noted the effect; and also had myself carefully observed by Dr. Cooper, in order that nothing might escape observation that would aid me in forming my conclusions. The urine was increased in quantity 3 or 4 ounces during the 24 hours, but the specific gravity was lowered 2 or 3 degrees as tested by the urinometer. Detected bromine in it within the first hour, but cannot say unhesitatingly that it was there in combination with potassium or whether with sodium, as Dr. Bill will have it. I think, however, that he is mistaken in regard to this as well as in other things in his article, and that we may regard it as being there in the form of bromide of potassium, but did not demonstrate the truth by experiments. I did not make a quantitative analysis of the urine, therefore am unable to produce anything that would be interesting on this subject. Dr. Bill has done so, but I do not think his observations are sufficiently reliable to justify us in adopting them without further investigation. I will, however, notice a few of them in this connection. The chlorides, he says, are greatly increased. Soda is increased and potassa diminished. Uric acid increased; urea not affected. Urine higher color. I did not find the latter true in any of my experiments, but on the contrary a diminution of color. He says further that the elimination of carbonic acid from the lungs is retarded, but its formation is not interfered with. He thinks that some vital cause is the reason for this diminished excretion, and that it is confined solely to the pulmonary mucous membrane. The soothing effect of the bromides he ascribes to the retained carbonic acid. He claims that a constipating effect is produced. I observed no special effect in this direction. I noticed that the appetite seemed keener for the first few meals; subsequently a peculiar change takes place. It is difficult to describe the change, however, for it cannot be said that a disrelish follows, but more of a carelessness in regard to eating, The sensation of hunger is not noticed; but when we sit to a meal it seems that as much can be eaten, yet without producing the usual feeling of satiety. Digestion is carried on apparently as well as ever, and no unpleasantness in the least follows a hearty meal. We conclude that the effect on digestion is to diminish the desire for food, the power for taking and assimilating remaining unaffected. The muscular system seemed relaxed and incapable of being used with the usual vivacity. I think, however, that this is not so much from the effect on the muscular system as upon its vital stimulus, the nervous system. I feel satisfied that the contractile property of muscle is but little, if at all, affected. We shall see more on this point as we progress. The cutaneous system is so affected that I am led to believe that it also affords a channel for elimination of the bromides. After one of the experiments, a very painful carbuncle came upon the back of my neck, which suppurated scarcely any. I cannot say that it had any connection with the taking of the bromide, yet I noticed at each subsequent trial that a peculiar little boil would show itself near the site of the old seat of trouble. But slight suppuration took place in any of them. There was a minute slough in each one, and the lymphatic glands were swollen and very painful in the region of the boils. I have observed this only on myself, and consequently cannot say that this was the result of the medicine. I believe, however, that these were from the effect of the large quantity taken, for the inflammations were peculiar, having never noticed anything like before; and they disappeared when I left off my experiments. But another affection presents itself as regularly as its use is persisted in. It is a minnte vesciculo-papular eruption, which causes an intense itching and which does not disappear for a considerable period after discontinuing the bromides. I infer from this that it finds exit through the skin, as well as the kidneys, and acts as an irritant to the sudoriferous glands.

During these experiments I became satisfied that the circulation was materially affected, and I made a special series of observations for the purpose of determining its real effect on temperature and circulation. The results I will produce further on. In regard to the effect on the nervous system, I must acknowledge the great difficulties to be overcome and the almost utter impossibility of being certain in the observations made. Even if one feels certain himself, he lacks the means of making it mechanically manifest, as can be done with the temperature, circulation, etc. Of all the systems we have yet noticed, the nervous was most impressed; indeed, I am of the opinion that this is the part primarily affected, but the results of the nerve change are variously manifested. The nerve tissue is endowed with certain properties which it will be best to notice before discussing the influence exerted on them. In common with other tissues, it contains the primary properties susceptibility and vital affinity; also properties peculiar to itself, impressibility and transmissibility. We have to do more especially with the last two named. The important relations which these properties sustain to the system at large make them objects of unusual interest, and need to be well understood. A slight impression on one or both of these properties has a great effect on the vital economy. Thus, through its receptivity or impressibility, the nervous system is made cognizant of the wants of the entire organism, and through the property of transmissibility it responds to the various calls upon it.

The functions of the nervous system are so various that it will be wholly impossible to notice them all. The receptivity of my nervous system was considerably diminished. Injuries were more easily borne, and the effects of heat and cold were not so easily noticed. Indeed, there was nothing that pertains to this function which was up to the normal grade of acuteness. As before noticed, the desire for food and drink appeared lessened, yet they were present, as the power of partaking and the relish felt for them will testify. A peculiar feeling of fullness of the head was present each time, but it was not an unpleasant sensation. No headache was present during any of the time, but on the contrary the usual causes failed to produce any. Study I could not, nor had I the faculty of concentrating my attention on anything. The best lecture would be but partly noticed. Memory for some reason was less reliable than normal, but I do not think it resulted from anything except the lack of ability to fix my thoughts. My feelings were what some would term pleasant, for I cared but little what transpired about me. The excito-motory nerves have their transmissibility diminished, which I think accounts for the muscular relaxation which was present. The sensory nerves are also diminished in their power of taking impressions. The secreto-nutritive nerves of Brown-Séquard I think are also more or less diminished in their function. I could not demonstrate this to my own satisfaction, however. I infer this more from the condition of my appetite and the long continued relaxation of habit, which remains after the bromides have been left off. Some claim that the reflex action of the cord is alone affected, but I am satisfied that the action is more general, as regards the nervous system. Its soporific effect has been greatly overestimated by some. I have never been able to find that it compels sleep like morphia. Still I have seen sleep follow its use, where morphia had failed entirely. It is true that when I was taking large quantities I could sleep when I pleased, and so long as I pleased; still I could, by an effort of the will, overcome the desire, and when asleep could be easily awakened. When I was awakened from such a sleep, my mind would be as clear as before sleep, and the desire to fall asleep again could be easily overcome. We see that it differs much from morphia and its allied products. They compel sleep, in spite of all opposition, when given in sufficiently large doses to produce their specific effects, while the bromides permit sleep. They calm the person if excited, or diminish his power for thought and for fixing the attention, and sleep follows as a consequence. Its anodyne properties have also been overestimated. This, like sleep, is only produced secondarily.

But the great point which prompts me to record these observations, and which I think will account for the action of the bromides better than any opinion yet produced by observers, remains to be noticed. I refer to the effect on circulation. I was unprepared, from my reading, to believe that such an effect was produced. All observers, nearly, either overlook or wholly deny that it has an effect on the circulation. One or two have hinted that some effect is produced, but have left it unexplained, so far as I know. Feeling certain that an effect was produced on the circulation, I procured a Marey's sphygmograph to see how it was affected. I also used an axillary thermometer to take observations on the temperature. I found out that the relation between large and small doses holds true in regard to circulation. In order that the result would be more manifest, I made use of pretty large doses, though not larger than are often given medicinally. My general health was good when I commenced the experiment which I will now record. My normal temperature is 98½°, pulse 78 to 80 per minute. I had noticed that my pulse was frequent for a considerable length of time, and waited for it to reach its normal frequency. Not appearing to change any and being normal in all respects except in frequency, I did not see fit to wait longer. I made other interesting observations, but will record but one in full.

This experiment was commenced on Jan. 1st, 1869, and was continued through four days. Temperature was taken under the tongue, by Teiman's thermometer graduated to ¼ degree. The following are the delineations:—

The amount which I took was ʒss., four times a day, for four days. The last four doses were bromide of ammonium. I took the first dose immediately after impression No. 1.

It is necessary to explain each of these separately, or mistakes are sure to be made. The sphygmograph delineates the volume and frequency, but the delineation of force is apt to mislead, and the deception can hardly be avoided except by a close watch through feeling the pulse, in addition to delineations, and noting carefully the condition under which each one was taken.

No. 1, which is an impression of the pulse unaffected by anything except ordinary exercise, shows a firm and a moderately full beat, with considerable frequency. If we compare the succeeding ones, taken during the effects of the bromides, we cannot fail to observe the difference. Nos. 4 and 16, when compared with No. 1, show a great difference.

The first eight were taken without removing the instrument, and extend through two hours and three-quarters; and we obtain very uniform results, especially with regard to frequency of pulse. The temperature appears to remain unaffected at first, but from prolonged use it becomes decidedly lowered. At first glance one might notice a want of uniformity in the results; still, as we shall see presently, the strictest uniformity prevails throughout. At first it has an apparent stimulating effect, as shown by numbers 1, 2, 3, and 4; but let us examine more closely. The volume of the pulse, it is true, is greatly increased, but it is at the expense of firmness. Instead of a firm, sharp stroke, we have now one which is soft and compressible. The amount of blood which passes by a given point, in a given time, is less in No. 4 than in No. 1. Thus we have at first the pulse diminished in force and frequency, and increased in volume. From No. 4 to No. 8 the force and frequency continue to diminish. No. 8 compared to No. 1 does not show a very great difference of delineation; yet they are impressions of very different conditions of the circulation. No. 8 is compressible and has a difference of 16 pulsations per minute. Numbers 11, 15, and 16 are those taken after a period of rest. They are exceedingly soft, especially Nos. 15 and 16. We notice quite a change in temperature towards the last, it having diminished from 100¼°, as shown by No. 1, to 97°, as shown by No. 16. Numbers 9, 10, and 13 show the effects of moderate exercise on the circulation, when compared with those taken after a period of rest; while Nos. 12 and 14 are impressions of pulse taken after violent exercise. They indicate a labored action of the heart, as it endeavors to supply the wants of the system, yet they too are soft and easily compressible. I thought it would not be safe nor discreet to carry the effects farther than is shown in No. 16. In order to see the effect of a diffusive stimulant in this condition, I took ar. spts. ammon. and tr. camph. āā ʒss. immediately after taking impression 16. Without having removed the instrument, I took impression 17 fifteen minutes after the stimulant. A very great change took place during this short time. The temperature raised one and one-half degree, while the pulse became quick, firmer, and irritable. This stimulating effect continued to increase for a considerable time. My head ached very severely as soon as I commenced getting under the influence of the stimulant, and continued aching until I took a good dose of bromide of ammonium. The subsequent delineations were of no unusual interest. They showed an increase in the force of circulation. My head never ached before, when I left off the bromide. I would gradually come out from under its influence, not feeling natural in less time than one week.

No one can say, after having carefully noticed these results, that circulation is not affected by the bromides of potassium or ammonium. I am aware that some will say that this will not be sufficient to base any conclusions upon. Anticipating this objection, I made various observations on different individuals, and the same result followed in all cases. This does not arise from any idiosyncrasy of mine, but is the uniform effect of the article in all cases. As regards preconceived notions, I can say that my conclusions are in no way affected by them. If I had any notions whatever they were all overturned in this investigation.

Now it is necessary to find out how this effect on the circulation is produced. I believed at first that the muscular coat of the arteries had its contractility diminished and that the effect was produced in this manner; but subsequently I became satisfied that if it was at all affected it was but to a slight degree. This I think is proved by the effect of stimulants and exercise, as shown in Nos. 12, 14, and 17. The vaso-motor nerves preside over circulation by bringing nervous influence to bear on the muscular fibre of the vessels. The walls of the vessels are highly elastic, and the contractility exactly corresponds to the dilatibility under ordinary circumstances. This property aids very greatly in aiding circulation. The arteries are distended by the vis a tergo of the heart, and the pressure of this blood affords a stimulus to the vascular coats causing them to contract, thereby sending the blood forward in its course. The heart is also endowed with the same property, but owing to its great power it will not succumb so readily as the arteries to agents which act upon them. Remembering these facts, it can readily be seen how such a condition of the circulation is produced. This can be produced in various ways: for instance, if the property of contractility is diminished, or the impressibility or irritability of the walls is lessened, or if the motor nerves have their function diminished, the same result will be produced.

In this case we have to do more with the nerves distributed to the vessels than with the vessels themselves. The power to respond to a stimulus remains in the arteries and heart, but the nervous supply is materially diminished in its function. The smaller arteries have much to do in the propulsion of the blood and in regulating the supply of blood to different parts of the system, by virtue of their muscular coat. Very many diseases are much influenced by this property of the arteries. In their normal condition the dilatation of their walls by the blood, which has been propelled forward by the heart, causes a corresponding contraction. This contraction differs from that which we find in the larger arteries and in the veins, on account of the muscular fibre contained in the coats of the smaller arteries.

The bromides change the action of these arteries, so that it more resembles the action of a vein, being merely a passive channel. We can see this beautifully shown in the first 8 delineations. No. 2 shows how soon the dilatabilility is increased, but the contractility is but little weakened. No. 4 is the last one which shows that contractility still acts equal to the dilation. After this we see a manifest lessening of the contractile power. During rest we see, as shown by No. 16, that the contraction is nearly absent, leaving the artery in an almost passive condition.

This will explain much of the efficacy of this article in disease, as we shall see further on. The surface of the body has a red flush all over, owing to the quantity of arterial blood which distends the smaller arteries and the capillaries. The difference between this condition and venous congestion is very marked; still the color is not that given by thoroughly oxygenated blood, owing to the tardiness of the circulation, which prevents it from being brought to the lungs sufficiently often. This causes a peculiar sense of fullness in the head, which is not at all unpleasant. I believe with Dr. Bill that there is also an excess of carbonic acid retained, still I do not believe as he does that the calmative property is owing wholly to this carbonic acid. I do not doubt that it has some effect in this direction, probably similar to that produced in an illy ventilated or crowded room.

In summing up the results of my investigations, I conclude: that it is the combination which causes the peculiar action, and not the bromine or the base; that it is quickly absorbed into the circulation and acts through the blood; that it is not decomposed to the extent claimed by Dr. Bill; that it produces an effect in a very short time after it is taken, and this effect is somewhat prolonged; that this effect is not dangerous within certain limits; that the primary effect is on the nervous system, diminishing all its functions somewhat, though more powerfully affecting its excito-motor and vaso-motor properties; and that through this influence on the nervous system the circulation, motion, sensation, mental action, etc., etc., are more or less affected, though not to an equal degree in each case.

With what we have learned of the action of the bromides in the healthy condition, we can more intelligently study the

"rationale" of their action in disease. Owing to the want of a proper knowledge in this respect, they are in danger of becoming unpopular, as too often happens with new remedies. They have been highly recommended in diseases where they can be of no earthly use, and even where they are contraindicated. By wisely discriminating, we shall find the bromides of great value in very many diseases. However, if given alone they are rarely curative like the iodide of potassium, etc., but act only as palliatives. We shall find them of great temporary benefit in all spasmodic affections, from terrible hydrophobia to the merest muscular twinge; also in all cases of nervous excitement, from maniacal delirium to the wakefulness of the hard thinker; but in no case will they act as curative except where the exciting cause is temporary. We should not rely on them wholly in such diseases as hydrophobia, puerperal convulsions, cholera, epilepsy, etc., where the cause will not be exhausted in a short time of itself; but to allay the spasmodic symptom of these diseases we have no article comparable to these; but in delirium tremens and those diseases in which the cause will soon become extinct we can rely on them alone. What has been said with regard to the muscular coat of arteries is equally true with regard to the muscular coats of the various ducts of the body; for instance the renal, the biliary, the ejaculatory ducts, etc.

We have far more potent anodynes than these, though they do blunt the sensibility to a certain degree. In this connection I will produce two sphygmographic delineations in order to show how a very irritable, quick pulse is affected. The subject was laboring under a subacute attack of pneumonia:—

No. 1.—Pulse, 102; temperature, 100° F.

No. 2.—Pulse, 120; temperature 100°, three hours after No. 1.

No. 1 was taken to see the condition unaffected. I then gave bromide of ammonium ℈ij. In three hours No. 2 was taken. No. 2 has lost the irritability present in No. 1, being soft, frequent, and compressible. The person felt no effect from the dose. It will be profitable to examine the manner of their action on a few special diseases. It has become very popular, and that with good reason, in the treatment of that terrible disease, epilepsy. Some have been greatly disappointed, however, but it resulted from not remembering or knowing that it has only a palliative action and that if the cause of the convulsions is not removed they will return so soon as the effects of the bromides have passed off.

Much difference of opinion has existed with regard to the pathology of this disease, and I think the opinion generally held at present is very much out of the way. It can be proved beyond a doubt, I think, that epileptiform convulsions of every variety are caused by a sudden anemia of the brain and spinal cord. There is a great variety of these convulsions, which are reducible to this class. Puerperal convulsions are but an attack of epilepsy under peculiar circumstances. Rigors, etc., are caused by anemia of the spinal cord. Now this anemia is caused by a spasm of the arterioles, distributed to the brain and cord.

We can easily see now how the convulsions and rigors are prevented by the bromides. In them we have nearly a specific for delirium tremens. In this disease we have the circulation irritable and the blood very deficient in nutrition. Now by retarding the flow of arterial blood it imparts to the brain more nourishment than it would if hurried, as it really is, during the excitement of the disease. Where it is superior to opium is in the fact that it causes arterial congestion, whereas opium causes venous congestion. We can easily see the advantage and find an explanation why it so often causes sleep in this disease when morphia has failed. It requires no explanation why it is useful in the passage of renal and biliary calculi; how it prevents seminal emissions, chordee, etc.; why spasmodic croup, asthma, whooping-cough and like diseases are so greatly relieved by the bromides.

How well I may have succeeded in convincing others of the truthfulness of these conclusions I cannot tell; but I feel satisfied in my own mind that they are the only views that have afforded a good explanation of the effects of the bromides in disease. I have been well repaid, by the satisfaction I derive from being now able to view their effects in a rational aspect. I do not think that I have claimed more for them than facts will allow. If this meets the approval of my medical brethren, well; but if it does not, all that I ask is that some one produce something better as an explanation, and I will be ready to give up these views. Until then, I can but regard these views as affording the true explanation of the action of the bromides.

## Figures and Tables

**No. 1. f1:**
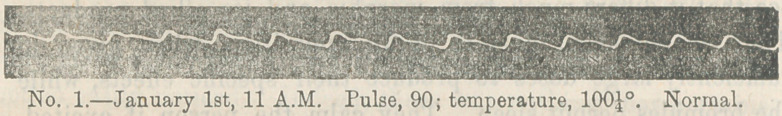


**No. 2. f2:**
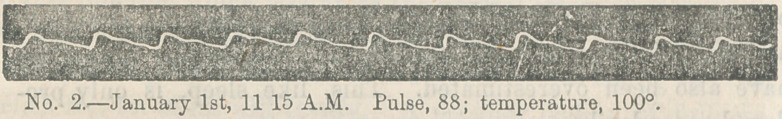


**No. 3. f3:**
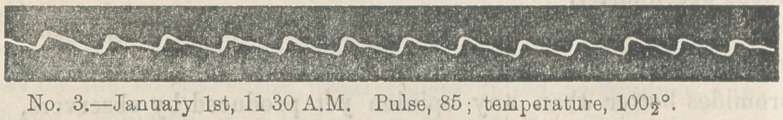


**No. 4. f4:**
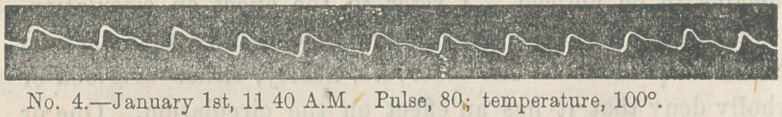


**No. 5. f5:**
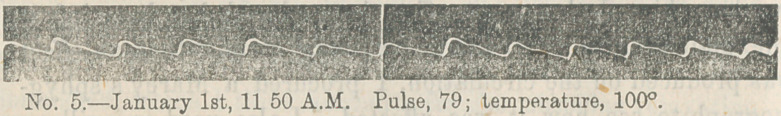


**No. 6. f6:**
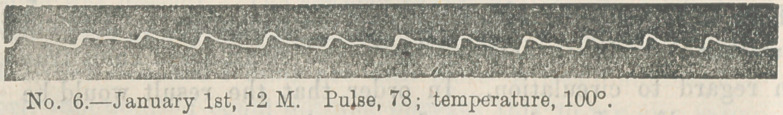


**No. 7. f7:**
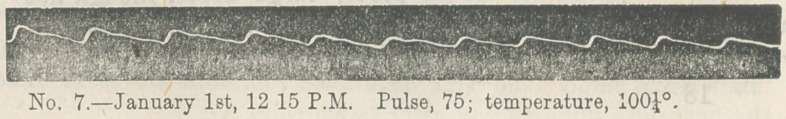


**No. 8. f8:**
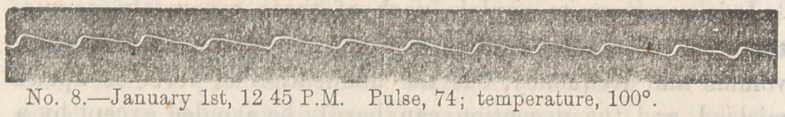


**No. 9. f9:**
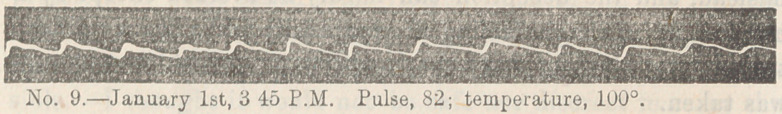


**No. 10. f10:**
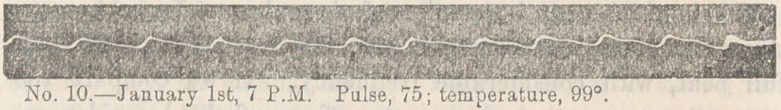


**No. 11. f11:**
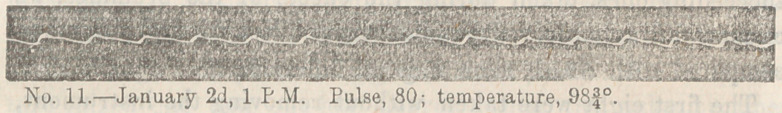


**No. 12. f12:**
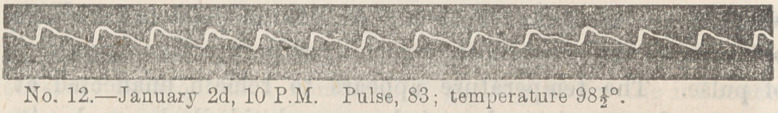


**No. 13. f13:**
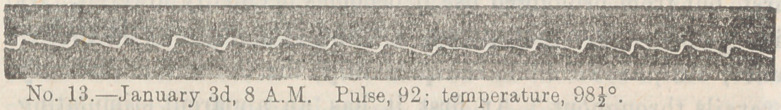


**No. 14. f14:**
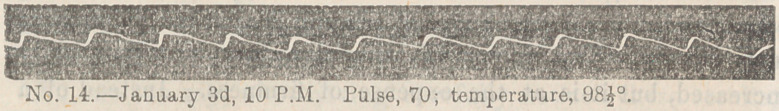


**No. 15. f15:**
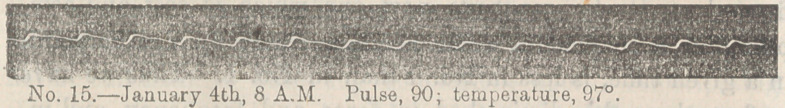


**No. 16. f16:**
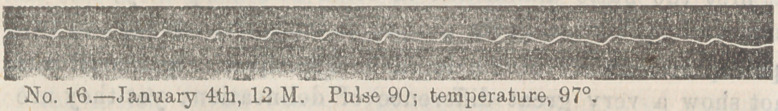


**No. 17. f17:**
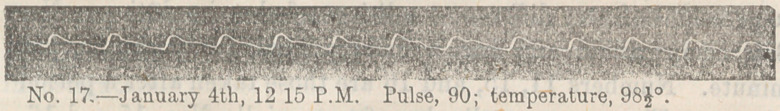


**No. 1. f18:**
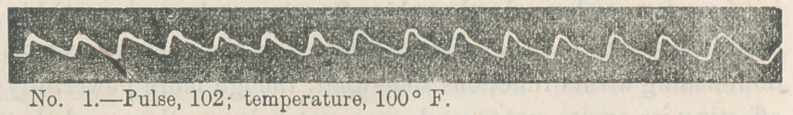


**No. 2. f19:**