# Correction

**Published:** 1869-07

**Authors:** 


CORRECTION.—In my article on "The Bromides," on page 275 of the EXAMINER, third line from the bottom, instead of ℥ss., it should be ʒss.

